# The Roles of Carbon‐Nitrogen Synergy and Phosphate Regulation in Producing Higher Yield of Vancomycin by *Amycolatopsis orientalis*

**DOI:** 10.1002/mbo3.70072

**Published:** 2025-10-29

**Authors:** Vineeth Mani, Sivakamavalli Jeyachandran, Mohammed Aman

**Affiliations:** ^1^ Department of Biotechnology and Bioinformatics Bishop Heber College Tiruchirappalli Tamil Nadu India; ^2^ Lab in Biotechnology and Biosignal Transduction, Department of Orthodontics, Saveetha Dental College and Hospital, Saveetha Institute Medical and Technical Sciences (SIMATS) Saveetha University Chennai Tamil Nadu India; ^3^ Department of Industrial Engineering, College of Engineering University of Business and Technology Jeddah Saudi Arabia

**Keywords:** *Amycolatopsis orientalis*, media optimization, production media, secondary metabolites, vancomycin

## Abstract

This study aimed to optimize culture media to enhance vancomycin production by *Amycolatopsis orientalis*. Using a combination of one‐variable‐at‐a‐time (OVAT) analysis, nutrient screening, and knockout experiments, we identified critical factors influencing biomass formation and antibiotic yield. Among tested carbon sources, maltodextrin significantly increased vancomycin production (bioassay zone: 13.33 mm), while glycerol favored biomass growth but had minimal impact on antibiotic synthesis. For nitrogen sources, soybean meal and soya flour optimally supported both biomass and vancomycin yield. Salt supplementation revealed that CaCO₃ and MgSO₄ improved mycelial growth, whereas knockout studies demonstrated that dextrose and soya peptone were essential for vancomycin production. Notably, omitting phosphate (K₂HPO₄) enhanced both biomass and antibiotic yield, suggesting phosphate repression of secondary metabolism. Seed media trials identified SS‐6 and SS‐10 + A as optimal for mycelial growth, highlighting the importance of early‐stage culture conditions for fermentation outcomes. These findings provide a cost‐effective, scalable strategy for maximizing vancomycin production, with potential for further optimization using statistical or metabolic flux approaches.

## Graphical abstract

Systematic media optimization in *Amycolatopsis orientalis* identified maltodextrin, soy‐based nitrogen sources, and phosphate regulation as key drivers of vancomycin yield. Knockout and feeding trials demonstrated essential nutrient roles, offering a cost‐effective, scalable strategy for enhanced antibiotic production.

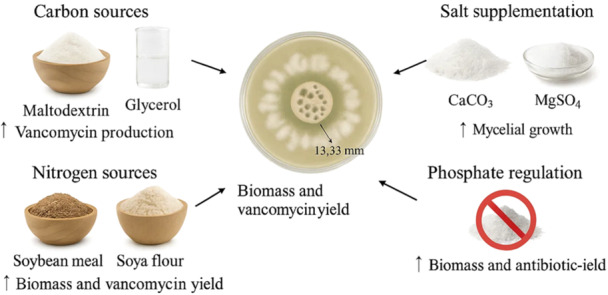

## 1 Introduction

Secondary metabolites constitute a diverse family of bioactive compounds formed by microbial, plant, or fungi during the late stationary growth phase. Unlike primary metabolites, these compounds have no direct role in growth or reproduction, but they perform ecological functions such as defense, signaling, and competitive survival (Bhatla and Lal 2023; Bérdy 2012). Of first descriptions to Albrecht Kossel in the late 19th century, secondary metabolites turned out to be crucial in the origin of pharmacological substances (antibiotics, immunosuppressors, anticancer drugs) (Demain and Sanchez 2009; Newman and Cragg 2020). *Actinomycetes*—genus *Amycolatopsis* have a well established reputation for the ability to produce glycopeptide antibiotics including vancomycin, last‐line option against multidrug resistant Gram‐positive pathogens (Kisil et al. 2021; Binda et al. 2014). Vancomycin biosynthesis in *A. orientalis* is tightly regulated by environmental and nutritional factors. The composition of culture medium amongst these makes a difference in intervening between growth and secondary metabolite production. Carbon and nitrogen sources, mineral salts, pH and aeration are able to substantially change the physiological condition of the organism and production of antibiotics (Banga et al. 2008; Jadon et al. 2019; Romero‐Rodríguez et al. 2018). Optimization of these parameters is not only necessary for maximum production, but also minimizing the fermentation costs which is highly relevant for the commercial scale biosynthesis. While, classical optimization strategies such as the one‐variable at a time (OVAT) approach have proven to be useful to increase medium composition, new advanced strategies, such as response surface methodology (RSM), artificial neural networks (ANN), genetic algorithms (GA) are more precise and scalable (Ibrahim and Abdul Wahab 2024; Elfghi 2016; Lahiri et al. 2021). However, empiricism still has benefits and can be used for initial screening and critical variable determination as long as jointed with factorial design and component knockout studies.

Although a lot of research has been done on vancomycin biosynthesis, there is still a knowledge gap as to what specific medium components and concentrations are optimum to support its production in *A. orientalis*. Previous works have highlighted the strain‐specific dynamics of nutrient consumption, highlighting the need for strain‐specific studies to determine the best carbon and nitrogen sources for a given producer strain (Temme et al. 2025; Howden et al. 2010). Additionally, the effect of trace elements and media morphology, in particular, during seed culture development, can drastically affect downstream fermentation productivity and antibiotic yield (van der Heul et al. 2018; Baltz 2016). The purpose of this study is a determination and enhancement of critical culture parameters that affect vancomycin synthesis in *A. orientalis*. By systematically screening carbon and nitrogen sources, seed media design, and key salt components, this study aims to improve antibiotic yield and establish a foundation for future scale‐up and metabolic engineering.

## 2 Materials and Methods

### 2.1 Microorganism

The vancomycin‐producing actinomycete strain, *A. orientalis* was procured in lyophilized form and used as the production organism in this study. *Bacillus subtilis*, used as the indicator strain for antibiotic bioassay, was isolated from a soil sample. Both organisms were maintained under appropriate culture conditions for experimental use (Shirling and Gottlieb 1966).

### 2.2 Culture Media

*A. orientalis* was initially cultured and maintained on multiple standard and production media, including Modified Medium 65 (M65), International Streptomyces Project Medium No. 2 (ISP2), Yeast Malt Dextrose Agar (YMD), International Streptomyces Project Medium No. 4 (ISP4), and Oatmeal Agar (OM) agar. The same base medium, CRM, was used for both seed development and as the basis for the production medium SS‐6. The effect of different carbon sources—including sorbitol, dextrose, fructose, maltodextrin, starch, mannitol, glycerol, and maltose—was tested in shaker flasks for vancomycin production. Similarly, the effect of various nitrogen sources—yeast extract, soya peptone, soybean meal, soya flour, tryptone, cottonseed flour, ammonium sulfate, potassium nitrate, and sodium nitrate—was evaluated. To assess the influence of carbon and nitrogen concentration, dextrose and soya flour were tested at a base concentration of 20 g/L and 10 g/L, respectively. Concentration ranges were adjusted through a hit‐and‐trial approach to determine optimal biomass and vancomycin yield (Jung et al. 2007; Jeyachandran and Ragavendran 2025).

### 2.3 Inoculum Preparation and Fermentation Conditions

Two flasks, each containing 25 mL of SS‐6 medium, were inoculated with a spore suspension to obtain a 42‐h vegetative inoculum. For fermentation trials, a 5‐L Electrolab reactor with a working volume of 3.35 L was used. The reactor was equipped with two six‐bladed disc‐turbine impellers (55 mm diameter). Agitation was maintained between 200 and 500 rpm Aeration was supplied at 1 vessel volume per minute (1 vvm), and the temperature was controlled at 28°C. Dissolved oxygen levels, monitored using an Ingold polarographic electrode, were not actively controlled but remained above 75% saturation throughout fermentation (Padma et al. 2002; Zhao et al. 2024).

### 2.4 Revival of A. Orientalis

The lyophilized *A. orientalis* culture was revived using sterile saline‐glycerol solution. The tip of the glass ampoule was flame‐heated and broken by adding sterile saline, allowing reconstitution for 5 min. A 0.1 mL portion of the reconstituted suspension was mixed with 0.1 mL sterile 50% glycerol in cryovials and stored at –80°C. A loopful of the revived suspension was streaked onto agar plates and incubated at 28°C for 10 days to assess colony development and sporulation.

### 2.5 Screening of Carbon and Nitrogen Sources

#### 2.5.1 Carbon Source Evaluation

Agar Plates: Media components were dissolved in 70 mL purified water with magnetic stirring. After pH adjustment, the volume was brought to 100 mL The medium was autoclaved at 122.5°C ±  1.5°C for 30 min, poured into sterile Petri dishes in a biosafety cabinet, and allowed to solidify.

Broth Media: Media components were weighed into a beaker and dissolved in 20 mL of water. A 25% yeast extract stock was autoclaved separately. After pH adjustment, the total volume was made up to 45 mL, distributed into two flasks, and autoclaved. Post‐sterilization, 3 mL of yeast extract was added aseptically to each flask.

Agar Method: Revived culture was first inoculated into SS2 and incubated at 28°C ±  1°C for 72 h. A 10% inoculum was then transferred into SS10 medium and incubated for another 72 h. A loopful of this culture was streaked onto plates containing individual carbon variants and incubated at 28  ±  1°C for 10 days. Plates were observed for colony growth, maturation, and sporulation patterns.

Broth Method: Each carbon variant flask was supplemented with 3 mL of 25% yeast extract, then inoculated with 10% of the SS10 culture. Cultures were incubated, and samples were taken at 48 and 72 h for pH measurement and wet cell weight (WCW) analysis. The WCW was determined by centrifuging the broth at 12,000 × g for 10 min and recording the pellet weight (Taiwo et al. 2011).

#### 2.5.2 Nitrogen Source Evaluation

Agar Plates: Nitrogen source media were prepared similarly to carbon media, with 70 mL of purified water used for initial mixing. After pH adjustment and volume completion to 100 mL, media were autoclaved and poured into sterile Petri plates.

Broth Media: Composition was dissolved in 40 mL water, pH adjusted, made up to 50 mL, and autoclaved. The broth was inoculated and analyzed similarly to carbon source screening.

Agar Method: A revived culture from an ultra‐low temperature (ULT) freezer was inoculated into SS‐2 and incubated for 72 h. A 10% inoculum was transferred to SS‐10 and incubated again for 70 h. The culture was streaked onto plates containing different nitrogen sources and incubated at 28°C ±  1°C for 10 days. Growth, maturation, and sporulation were recorded.

Broth Method: Each nitrogen variant broth was inoculated with 10% of the SS‐10 culture. Samples were collected at 24 and 48 h for pH and WCW analysis using standard methods (McIntyre et al. 1996).

### 2.6 Media Preparation Strategy

All chemicals were weighed according to required composition and dissolved in 70 mL of purified water. After stirring and pH adjustment, the total volume was made up to 100 mL. The medium was autoclaved at 122.5°C ±  2.5°C for 20 min. For agar media, molten media were poured into Petri dishes in a biosafety cabinet and stored at 2°C–6°C for up to 15 days. Broth media were used immediately after sterilization.

### 2.7 Determination of Vancomycin Production

Vancomycin production was quantified using the agar well diffusion assay against *Bacillus subtilis*, as described by Bisicchia et al. (2011). LB agar was seeded with *B. subtilis*, and wells were made using a sterile rod. Twenty microliters of cell‐free culture supernatant were added to each well. Plates were incubated at 37°C for 16 h, and zones of inhibition were measured in millimeters. Standard vancomycin solutions of known concentrations were used as controls.

### 2.8 Estimation of Wet Cell Weight (WCW)

To determine WCW, broth cultures were transferred into pre‐weighed falcon tubes. The tubes were weighed again, centrifuged at 12,000 × g for 10 min, and the pellet weight recorded. The WCW was calculated using the formula:

WCW=(W3−W1)/(W2−W1)×100,

where

*W*1 = weight of empty falcon tube.

*W*2 = weight of falcon + broth.

*W*3 = weight of falcon + pellet.

### 2.9 Instruments

Vancomycin analysis was carried out by HPLC method. The Stationary phase [Column]: End capped ODS (C18 column) and the Mobile phase: ACN: Water [45:55]. The sample Run time: 15 min coupled with a UV detector (Detector Shimadzu SPD M28) at the absorbance at 229 nm.

### 2.10 Morphological Observation

Mycelial morphology of *A. orientalis* was examined using light microscopy. Samples were prepared by pipetting a small amount of culture onto glass slides, followed by Gram staining according to the standardized procedure (Zhao et al. 2024).

### 2.11 Statistical Analysis

All experiments were performed in triplicate, and data are presented as mean ± standard deviation (SD). Statistical comparisons of multiple treatment groups (e.g., carbon and nitrogen source screening, seed media trials, knockout studies, and feeding experiments) were carried out using one‐way analysis of variance (ANOVA). For experiments involving two independent variables (e.g., carbon–nitrogen concentration variations), two‐way ANOVA was applied. Post‐hoc multiple comparison tests (Tukey's HSD) were used to identify significant differences between groups. A *p*‐value < 0.05 was considered statistically significant. All analyses were performed using GraphPad Prism version 9.5.0 (GraphPad Software, San Diego, USA).

## 3 Results

### 3.1 Revival of Culture

**Figure 1:**
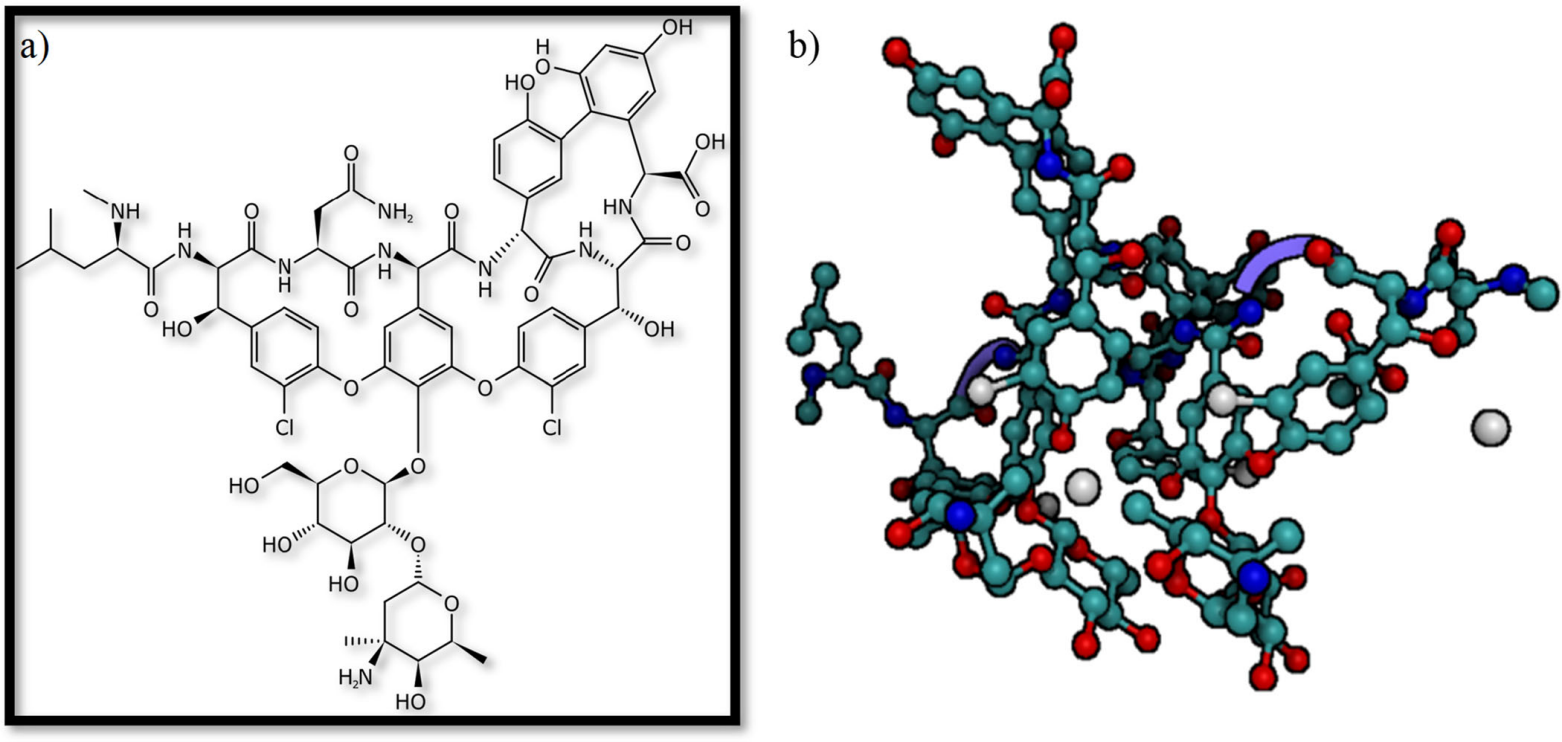
(a) Vancomycin chemical structure. (b) Crystal structure resolved by XRD (1.09 Å).

*A. orientalis* was recovered on a number of agar media (ISP2, ISP4, M65, YMD and OM). Among these; YMD, OM and M65 showed better colony growth and spore form characteristics (Figure 1a,b). These media formed volcano‐shaped, wavy colonies with considerable spores within the 10th day. Quantitative colony counts confirmed that YMD plates yielded significantly higher spore density (142 ± 8 colonies/plate) compared to ISP2 and ISP4 (*p* < 0.05, one‐way ANOVA). As a result, cell banks were prepared from the plates and used for further testing. The morphology of colonies and spore development are summarized in Supporting Information: Table S1.

### 3.2 Carbon Source Screening

**Figure 2:**
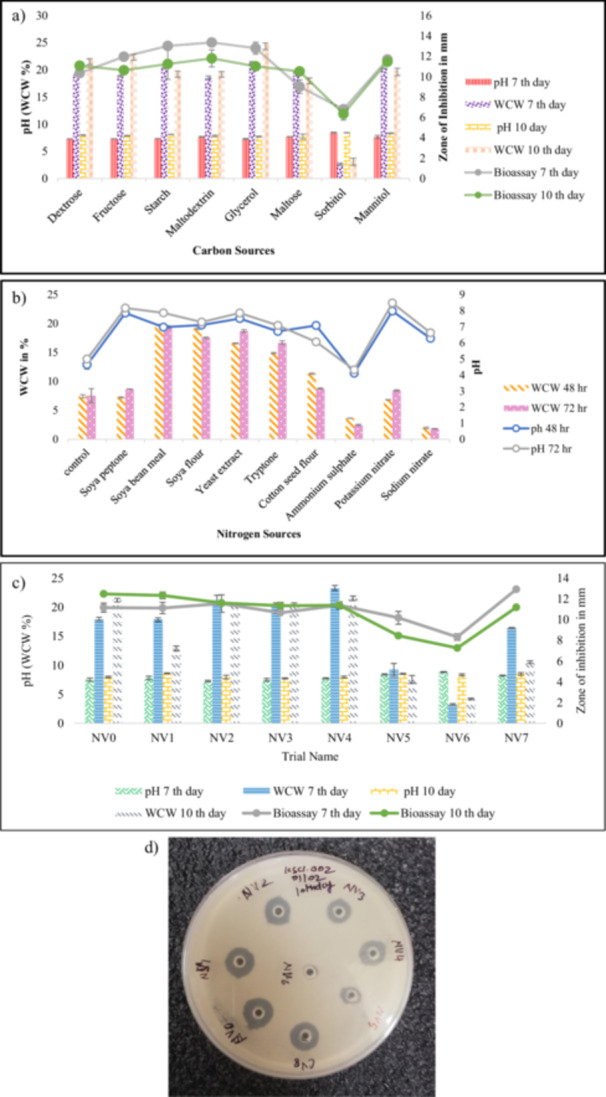
(a) Screening of carbon sources for biomass (WCW%), pH, and zone of inhibition (ZOI) on days 7 and 10. (b) Screening of Different Nitrogen sources for the biomass build up (WCW%) and pH trend 48 h and 72 h. (c) Effect of variation in Carbon and nitrogen source concentration in SS‐6 media for Production of Vancomycin, pH, and biomass build‐up (WCW%) on 7th and 10th days sample. (d) Bioassay plates of variation in carbon and nitrogen source concentration in SS‐6 media for the design of production media selection.

Carbon plays an important role in biomass accumulation and antibiotic activity in heterotrophs. To determine the best carbon source for vancomycin biosynthesis, *A. orientalis* was grown in media enriched with sorbitol, dextrose, fructose, maltodextrin, starch, mannitol, glycerol, and maltose (Figure 2a). Maltodextrin among these supported the highest zone of inhibition in bioassay (13.33 ± 0.42 mm) while that of glycerol yielded highest wet cell weight (WCW) of 24.67% ± 0.61%. However, glycerol was excluded in further fermentation trials because of its poor vancomycin production performance. Dextrose and starch produced intermediate bioassay zones (11.42 ± 0.38 mm and 10.71 ± 0.29 mm, respectively). The rapid consumption of some of the carbon sources (sorbitol, mannitol, fructose, glycerol and maltose) may constrain carbon supply available for secondary metabolism (< 8 mm; *p* < 0.05). The patterns of physical growth observed with various carbon sources are shown in Supporting Information S1: Table S2.

### 3.3 Nitrogen Source Screening

Nitrogen is a vital constituent of microbial synthesis of primary and secondary metabolites such as antibiotics. *A. orientalis* was grown in medias with different types of organic and inorganic nitrogen sources (yeast extract, soya peptone, soybean meal, soya flour, tryptone, cottonseed flour, ammonium sulfate, potassium nitrate, and sodium nitrate) (Figure 2b). Soybean meal, soya flour, and yeast extract had the WCW values 19.81% ± 0.65%, 19.10% ± 0.72% and 18.71% ± 0.58% respectively, which measured their ability to enhance biomass. Other sources such as tryptone and ammonium salts for example did not serve this purpose very well (Supporting Information S1: Table S3).

### 3.4 Effects of Carbon and Nitrogen Concentration Variations in SS‐6 Medium

After the SS‐6 medium had been selected, efforts were made to determine the effect of modification of the concentration of carbon and nitrogen sources on biomass and antibiotic yield. The highest WCW (23.30% ± 0.48%) was achieved with the use of NV4, and the highest bioassay zone (12.92 ± 0.36 mm) followed the use of NV7 (Figure 2c,d). These values were significantly greater than the control SS‐6 medium (21.21% ± 0.45% WCW; 12.48 ± 0.31 mm; *p* < 0.05).

### 3.5 Screening of Seed Media

**Figure 3:**
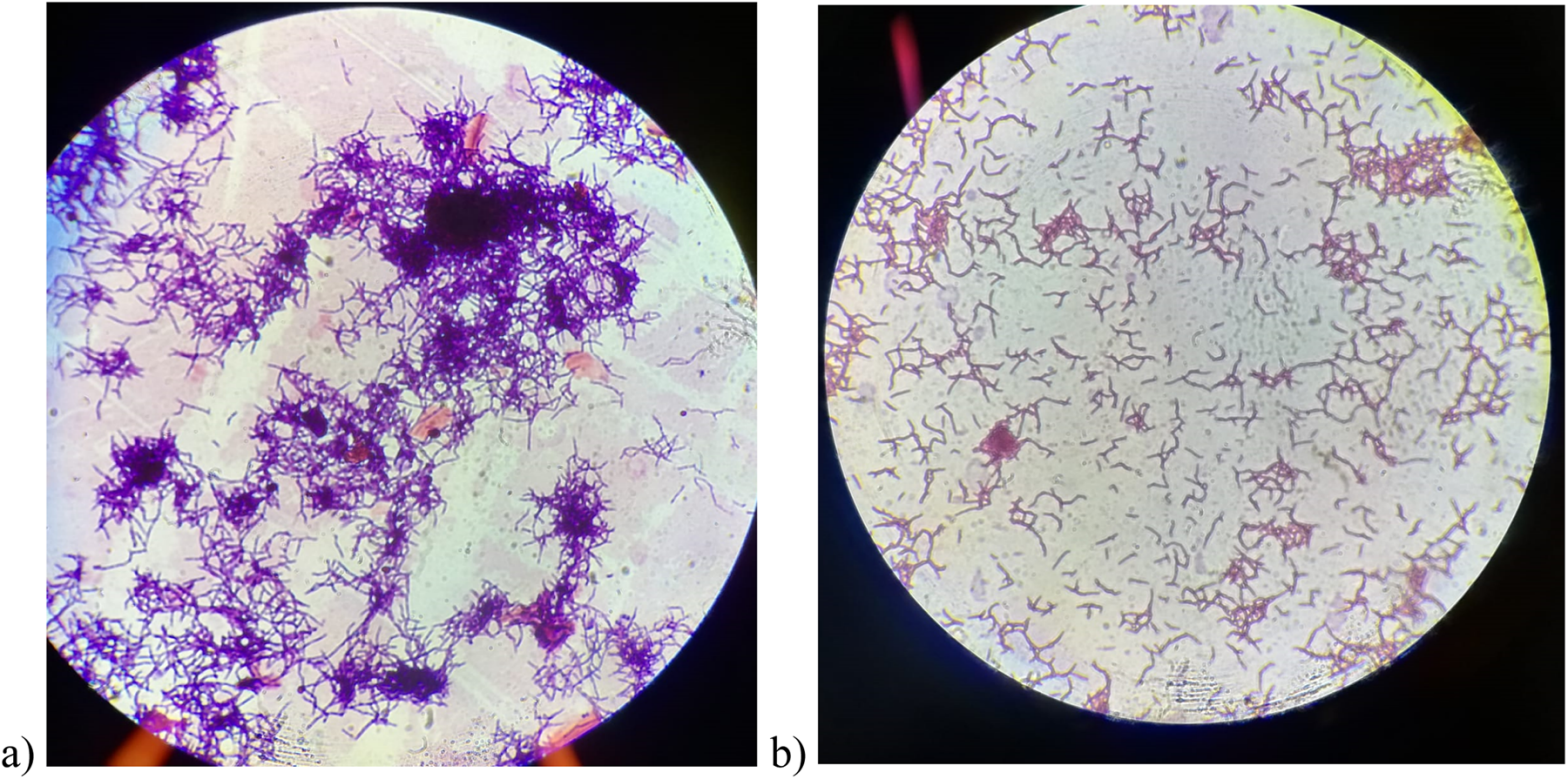
(a) Pelleted and (b) dispersed mycelial growth morphologies.

**Figure 4:**
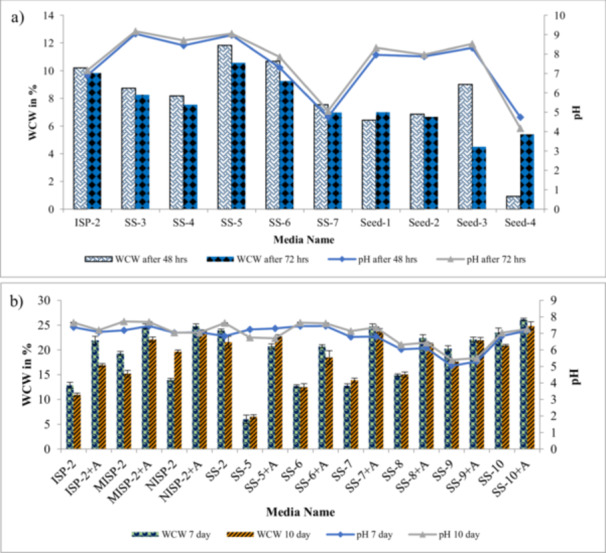
(a) Screening of various seed media for biomass build‐up and check pH trend 48 h and 72 h sample. (b) Screening of various media combinations with and without agar for mycelia growth, pH, and biomass build‐up 7th and 10th days sample.

The role of seed media is significant with biomass growth for inoculation. Several types of seed media were put to test to sediment mycelia growth (Supporting Information S1: Table S4). Among these, ISP‐2, SS‐5, and SS‐6 exhibited superior biomass formation with visible pellet formation and color differences (Figure 3a,b). To prevent pellet formation and facilitate mycelial dispersal, other seed media containing 0.2% of agar were tried. SS‐2 and SS‐10 + A yielded promising results with regard to dispersed mycelial growth and pH trends (Figure 4a,b). Further knock out experiments on SS‐2 validated the role of various components of the media in WCW and mycelial morphology fermenter sample.

### 3.6 Knockout Trial

**Figure 5:**
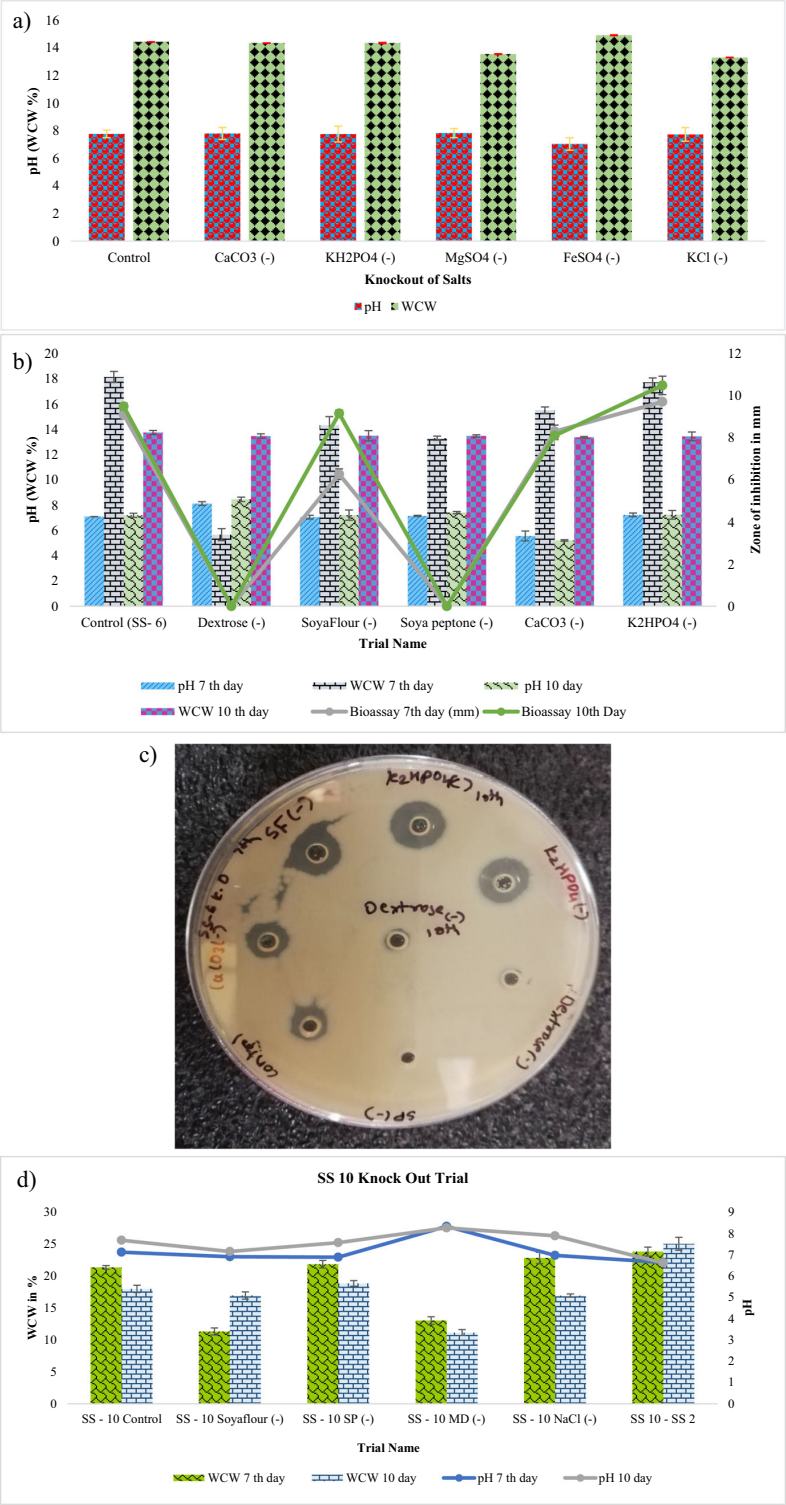
(a) Knockout of different salt from CRM media and see the effect on WCW, pH, and mycelial growth 48 h sample. (b) Effects of component knockouts in SS‐6 medium on vancomycin production (bioassay), pH, and WCW (days 7 and 10. (c) Bioassay plate of Knockout trial SS‐6 media after 10th day incubation sample used the ZOI is measured mm. (d) Knock‐out of different components from SS‐10 media for WCW%, pH 7th and 10th days sample.

The knockout method was developed by removing one component from the control media. The knockout trial study helps to find the particular component effects of microorganism growth and secondary metabolite (antibiotics). In this experiment, we checked the effect of different salt on CRM media. In SS‐10 media, we checked the effect of carbon, nitrogen, and salt. As shown in Figure 5a, while removing FeSO_4_ WCW weight increased meanwhile in the SS‐10 knockout trial seen the effect of soya flour and maltodextrin on WCW was drastically decreased. Knockout trial helps reduce the cost of media and increase product synthesis. In the SS‐6 knockout trial when removing components. In the absence of Dextrose and Soya–peptone no vancomycin was seen in the bioassay.

#### 3.6.1 CRM Salt Knockout Trial

In CRM media, different salts are used such as CaCO_3_, KH2PO_4_, MgSO_4_, FeSO_4_, and KCl to knock out one salt from each set of flasks and then observe the effect of growth. As mentioned in Figure 5a, there was not much difference in WCW but Omission of FeSO₄ significantly enhanced biomass, better mycelial growth and a slight increase in WCW were noted. No significant difference was observed for omission of CaCO₃, MgSO₄, or KCl (*p* > 0.05).

#### 3.6.2 SS‐6 Knock‐Out Trial

The Knockout from SS‐6 media one component from each flask then incubation for 10 days. The results of WCW value and bioassay analysis show that without dextrose and soya peptone no bioassay result on plates as shown in Figure 5b. It signifies that dextrose and soya peptone has major effect on antibiotic activity. In contrast, omission of phosphate (K₂HPO₄) significantly increased both biomass (17.74% ± 0.55% WCW) and antibiotic activity (10.49 ± 0.29 mm) compared to control SS‐6 (*p* < 0.05). The major finding of this trial is K_2_HPO_4_, dextrose, and soya peptone and how it affects the growth and vancomycin production as shown in Figure 5b,c (Supporting Information S1: Figure S1).

#### 3.6.3 SS‐ 10 Knock‐Out Trial

Removal of maltodextrin from SS‐10 medium resulted in a drastic reduction in biomass (11.28% ± 0.46% WCW) compared to the complete medium (19.62% ± 0.61% WCW; *p* < 0.001), confirming its essential role as shown in Figure 5d.

### 3.7 Effect of Feeding Selected Amino Acids (Phenylalanine and Tyrosine) on the Production of Vancomycin

**Figure 6:**
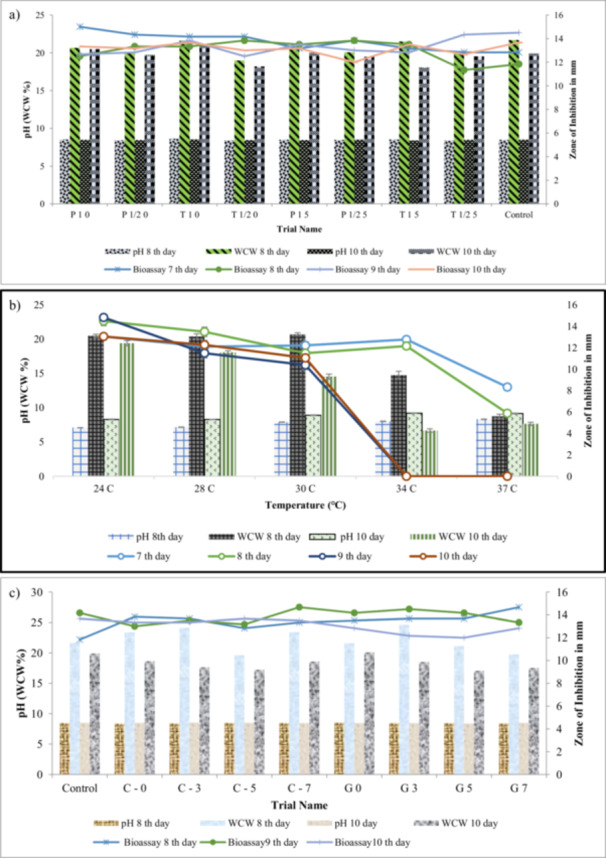
(a) Effect of feeding selected amino acids… for Bioassay (ZOI), pH, WCW%, of 7th, 8th, 9th, and 10th days sample used. (b) Effect of Temperature for screening biomass build‐up (WCW), pH, and production of vancomycin by checking with Bioassay (ZOI) on 7th, 8th, 9th, and 10th days sample used at 24, 28, 30, 34, and 37°C. (c) Effect of Feeding of Glucose and Citric acid on formation of biomass and production of vancomycin, feed feeding helps in production of vancomycin as mention bioassay of C‐7 in graph, 8th 9th, and 10th days sample was used to check the trend of pH, WCW%, and Bioassay.

In this trial, the amino acid l‐phenylalanine, and tyrosine were checked at different concentrations by feeding method in SS‐6 media on respective days: 3rd, 4th 5th also identified as positive variables. As shown in Figure 6a, at P10 the highest bioassay result of 15 mm on 7th day was observed. In contrast, feeding with l‐asparagine or glutamate reduced bioassay zones to below 11 mm (*p* < 0.05).

### 3.8 Optimization Incubation Temperature

Temperature plays a vital role in antibiotics production and biomass build‐up. Using several incubation temperatures ranging from 24°C to 37°C, the optimal temperature for antibiotic synthesis was observed. The pH 6.8 media was cultured for 10 days in a shaking incubator at 180 rpm, in triplicate for each temperature. After checking the bioassay as mentioned **in** Figure 6b; bioassay result was highest at 24°C.

### 3.9 Effect of Feeding of Glucose and Citric Acid on Formation of Biomass and Production of Vancomycin

The correlation between vancomycin antibiotic production and intra‐extracellular TCA intermediate products, alpha‐ketoglutarate dehydrogenase, and glyoxalate shunt isocitrate lyase activities, all of which play important roles in carbon metabolism, was investigated depending on the glucose concentration of *A. orientalis* medium with respect to incubation. Supplementation with glucose and citric acid significantly enhanced vancomycin yield compared to un‐supplemented control (*p* < 0.05). At day 9, glucose‐fed cultures showed inhibition zones of 14.28 ± 0.36 mm, while citric acid feeding yielded 13.94 ± 0.33 mm, both significantly higher than control (11.82 ± 0.29 mm).

### 3.10 Fermentation

**Figure 7:**
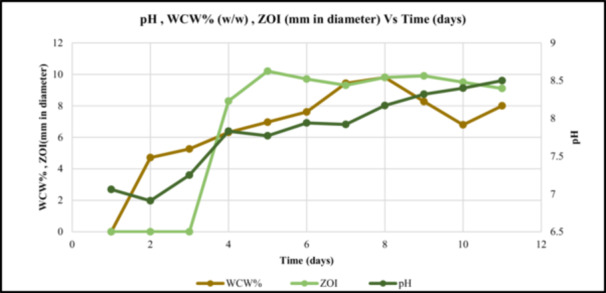
A fermentation time course graph vs WCW %, ZOI in mm, and pH, the sample used for analysis incubation time was 1st to 10th day, The ZOI was highest on the 5th day sample, that is, 10.2 mm and WCW 9.8% on the 8th day, and pH gradually decrease every day.

*A. orientali*s has grown in CRM media and then inoculate 1% into SS‐6 seed media. A 2% vegetative seed was used to inoculate the fermentation medium which is also SS‐6. The medium was adjusted to pH 6.8 with HCl/NaOH before sterilization. A fermentation time course graph vs WCW %, ZOI in mm, and pH graph drew. The ZOI was highest on the 5th day sample, that is, 10.2 ± 0.31 mm (*p* < 0.05 compared to days 4 and 6) and WCW 9.8% ± 0.27% on the 8th day, and pH gradually increased every day. The ZOI data get with bioassay analysis done of each day and compared with blank and standard at different concentration from 50 to 500 µg/mL as shown in Figure 7. The amount of the antibiotic produced by the fermentation was measured by high‐performance liquid chromatography (HPLC) under the conditions mention in HPLC analysis.

### 3.11 HPLC

The vancomycin content was analyzed by HPLC coupled with a UV detector (Detector Shimadzu SPD M28) at the absorbance at 229 nm. 5 μm C18 column (4.6 × 250 mm, 100 Å) was employed with a column temperature of 25°C. the mobile phase: ACN: Water [45:55]. The sample Run time: 15 min and the pH was adjusted to 3.2–3.3 with phosphoric acid. The procedure was based on samples were pretreated to reduce the abundance of proteins, lipids, and different metabolites in the culturing broth. Gradient elution was employed to separate similar‐polarity compounds. The mixture was chilled at 4°C for 20 min to precipitate proteins and then centrifuged at 10,000 rpm for 10 min. The gradient elution mode used a gradual increase from 0% to 30% of acetonitrile in the first 5 min, then returned to 100% of phosphate buffer in the next 3 min. The flow rate was maintained at 1 mL/min (Supporting Information S1: Figure S2).

## 4 Discussion

The present study highlights the pivotal role of media optimization in enhancing vancomycin biosynthesis by *A. orientalis*, demonstrating how carbon, nitrogen, salt composition, and seed media collectively shape fermentation outcomes. The results underscore the secondary nature of vancomycin production, where factors that favor biomass accumulation do not necessarily align with those maximizing antibiotic yield. Among carbon sources, maltodextrin emerged as the most effective enhancer of vancomycin production, generating a 13.33 mm inhibition zone, while glycerol primarily supported biomass without significantly improving antibiotic yield. This observation supports the long‐standing view that slowly metabolized carbohydrates, such as maltodextrin, prolong the idiophase, thereby maintaining metabolic flux toward secondary metabolite pathways (Minas 2005). Comparable findings have been documented for erythromycin in *Saccharopolyspora erythraea* (Mehanna et al. 2019) and avermectin in *S. avermitilis* (Chen et al. 2008; Dhandapani et al. 2022), where maltodextrin extended carbon availability and stimulated sustained biosynthesis. In contrast, rapidly metabolized sugars such as fructose and mannitol were associated with carbon exhaustion and diminished metabolite accumulation, suggesting that balanced substrate uptake is essential for prolonged antibiotic production (B et al. 2025; V et al. 2023).

Nitrogen source trials reinforced the advantage of complex organic nitrogen, with soybean meal and soya flour producing the highest wet cell weight and vancomycin activity. Complex nitrogen sources supply amino acids, peptides, and growth factors that likely enhance both protein synthesis and regulatory signaling. Previous studies on rapamycin production in *S. hygroscopicus* (Mohamed et al. 2019; Sohoni et al. 2014) and teicoplanin in *Actinoplanes teichomyceticus* (Jung et al. 2007) similarly demonstrated superior yields with soy‐derived components compared to inorganic salts (Wang et al. 2014). This emphasizes that nitrogen availability not only fulfills a nutritional role but also modulates global transcriptional programs governing secondary metabolism. Salt composition also contributed significantly to fermentation outcomes. The supplementation of CaCO₃ and MgSO₄ improved both mycelial morphology and WCW, consistent with their established roles as buffering agents and enzyme cofactors (Singh et al. 2012). Knockout studies revealed that FeSO₄ omission enhanced mycelial growth, suggesting that excess iron may impose oxidative stress or interfere with enzyme regulation (Chen et al. 2025). The most striking finding, however, was that phosphate (K₂HPO₄) omission increased both biomass and vancomycin production, indicating the presence of phosphate repression.

Phosphate control is a well‐documented regulatory mechanism in actinomycetes, mediated predominantly by the two‐component PhoR–PhoP system. In *S. coelicolor*, phosphate excess represses actinorhodin and undecylprodigiosin production by directly downregulating biosynthetic gene clusters (Yao et al. 2023). Similarly, phosphate limitation has been shown to derepress antibiotic biosynthesis in *S. griseus* and *S. lividans* (Le and Siriwatwechakul 2021). The mechanism involves PhoP binding to PHO boxes in promoter regions of both primary metabolism and secondary metabolite pathways, shifting transcriptional priorities toward growth when phosphate is abundant. In the context of *A. orientalis*, the repression observed here likely reflects conserved phosphate regulatory circuits that suppress vancomycin biosynthetic genes under phosphate‐rich conditions. Thus, fine‐tuning phosphate availability emerges as a crucial strategy for derepressing vancomycin biosynthesis and maximizing yields. Seed media trials further demonstrated that early‐stage morphological characteristics strongly influence downstream fermentation performance (Krysenko and Wohlleben 2024). Dispersed mycelial growth, as achieved with SS‐6 and SS‐10 + A, facilitated superior inoculum quality compared to pellet‐dominated morphologies. Similar effects have been reported in streptomycin production by *S. lavendulae*, where mycelial dispersal correlated with higher antibiotic yields (Donald et al. 2022). The ability to manipulate morphology through media design offers a practical approach for improving inoculum performance in large‐scale fermentation.

The integrative approach of combining OVAT screening with knockout trials in this study enabled precise identification of essential and non‐essential media components. The absolute requirement of dextrose and soya peptone for vancomycin production aligns with previous findings in *S. clavuligerus*, where omission of core carbon or nitrogen components abolished clavulanic acid biosynthesis. This highlights the interdependence of basal carbon–nitrogen synergy and secondary metabolism initiation. Overall, this study not only validates the central roles of carbon, nitrogen, and mineral salts in vancomycin biosynthesis but also reveals the importance of phosphate regulation as a molecular checkpoint. Future directions may involve integrating transcriptomic or proteomic approaches to map phosphate‐responsive regulatory circuits in *A. orientalis*. Additionally, statistical tools such as response surface methodology (RSM) could refine media optimization (Lahiri et al. 2021), while metabolic engineering of PhoP/PhoR or associated signaling pathways may enable stable derepression of vancomycin biosynthesis under controlled fermentation conditions. Together, these strategies provide a foundation for both academic investigation and industrial application, addressing the urgent demand for scalable production of this critical glycopeptide antibiotic.

## 5 Conclusion

This study demonstrates the critical role of strategic media optimization in enhancing vancomycin production by *A. orientalis*. Through systematic screening, maltodextrin and soy‐based nitrogen sources (soybean meal, soya flour) were identified as key drivers of antibiotic yield and biomass accumulation, respectively. Salt supplementation with CaCO_3_ and MgSO₄ further improved mycelial growth, while knockout experiments revealed the essential roles of dextrose and soya peptone in vancomycin biosynthesis. Notably, phosphate (K₂HPO₄) omission enhanced production, suggesting its repressive effect on secondary metabolism. Seed media optimization, particularly SS‐6 and SS‐10 + A, proved vital for achieving dispersed mycelial growth and optimal fermentation performance. These findings provide a practical, cost‐effective framework for maximizing vancomycin yields, with immediate applications in lab‐scale production. Future studies could integrate statistical modeling or metabolic engineering to further streamline industrial‐scale biosynthesis, addressing global demands for this critical antibiotic.

## Author Contributions

**Sivakamavalli Jeyachandran:** conceptualization, investigation, resources, data curation, writing – original draft preparation, writing – review and editing, supervision, project administration, funding acquisition. **Vineeth Mani:** methodology, software, investigation, data curation, writing – original draft preparation, visualization. **Mohammed Aman:** validation, formal analysis, resources, writing – review and editing, supervision, funding acquisition. All authors have read and agreed to the published version of the manuscript.

## Ethics Statement

The authors have nothing to report.

## Conflicts of Interest

The authors declare no conflicts of interest.

## Supporting information


**Supplementary Figure 1:** Bioassay plate of fermenter sample: A (0‐62 hrs), B (87‐136 hrs), C (138‐182 hrs), and D (230 hrs) with blank and Standard at different concentration from 50‐500ug/mL. **Supplementary Figure 2:** HPLC analysis of A) Vancomycin STD 0.1mg/ml B) ISP4 CB SS69th day C) SS‐10 CB SS6 9^th^ day and D) YMDCB SS6 9^th^ day.

**Supplementary Table 1:** Physical characteristics of *A. orientalis* in different types of media for spore growth. **Supplementary Table 2:** Physical characteristics of *A. orientalis* in different carbon sources. **Supplementary Table 2:** Physical characteristics of *A. orientalis* in different Nitrogen sources. **Supplementary Table 4**: Physical characteristics of *A. orientalis* in different seed media.

## Data Availability

Data available on request due to privacy/ethical restrictions.
