# Supplementary material for: The Roles of Carbon‐Nitrogen Synergy and Phosphate Regulation in Producing Higher Yield of Vancomycin by Amycolatopsis orientalis
Source: Microbiologyopen. 2025 Oct 29;14(6):e70072. doi: 10.1002/mbo3.70072 (PMC12569529; doi:10.1002/mbo3.70072)
Supplement: Supplementary file 1 — Supplementary Figure 1: Bioassay plate of fermenter sample: A (0‐62 hrs), B (87‐136 hrs), C (138‐182 hrs), and D (230 hrs) with blank and Standard at different concentration from 50‐500ug/mL. Supplementary Figure 2: HPLC analysis of A) Vancomycin STD 0.1mg/ml B) ISP4 CB SS69th day C) SS‐10 CB SS6 9th day and D) YMDCB SS6 9th day. [file MBO3-14-e70072-s002.docx]

**
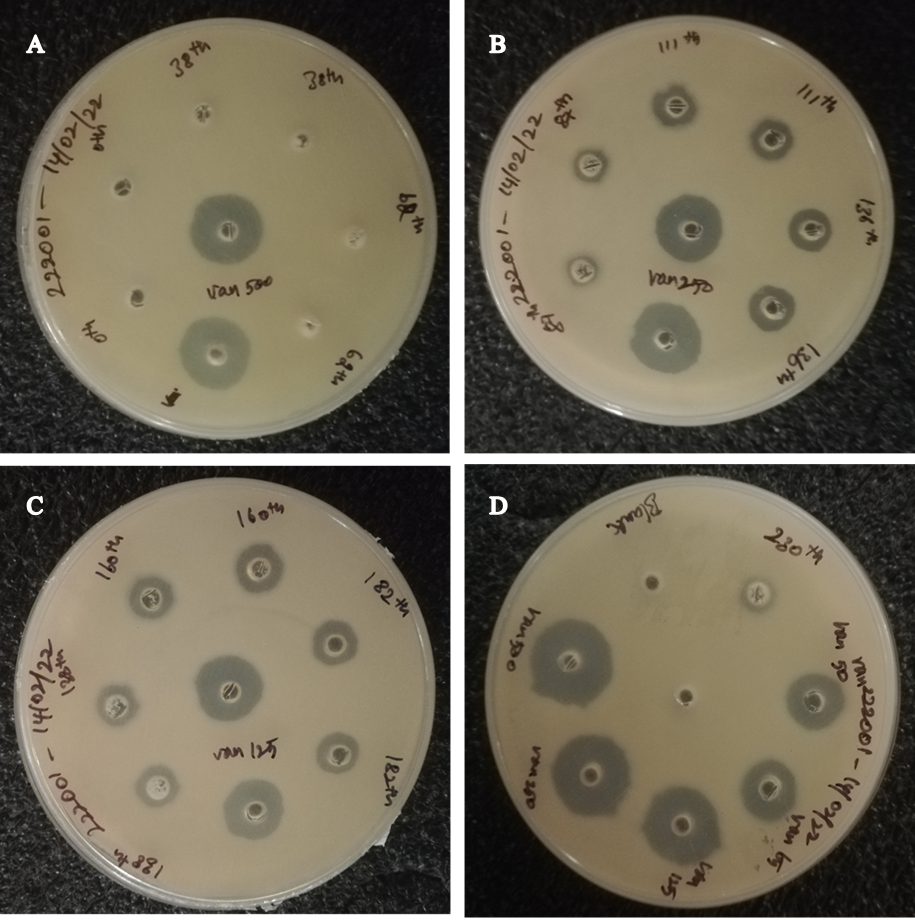
**

**Supplementary Figure 1.** Bioassay plate of fermenter sample: A (0-62 hrs), B (87-136 hrs), C (138-182 hrs), and D (230 hrs) with blank and Standard at different concentration from 50-500ug/mL.


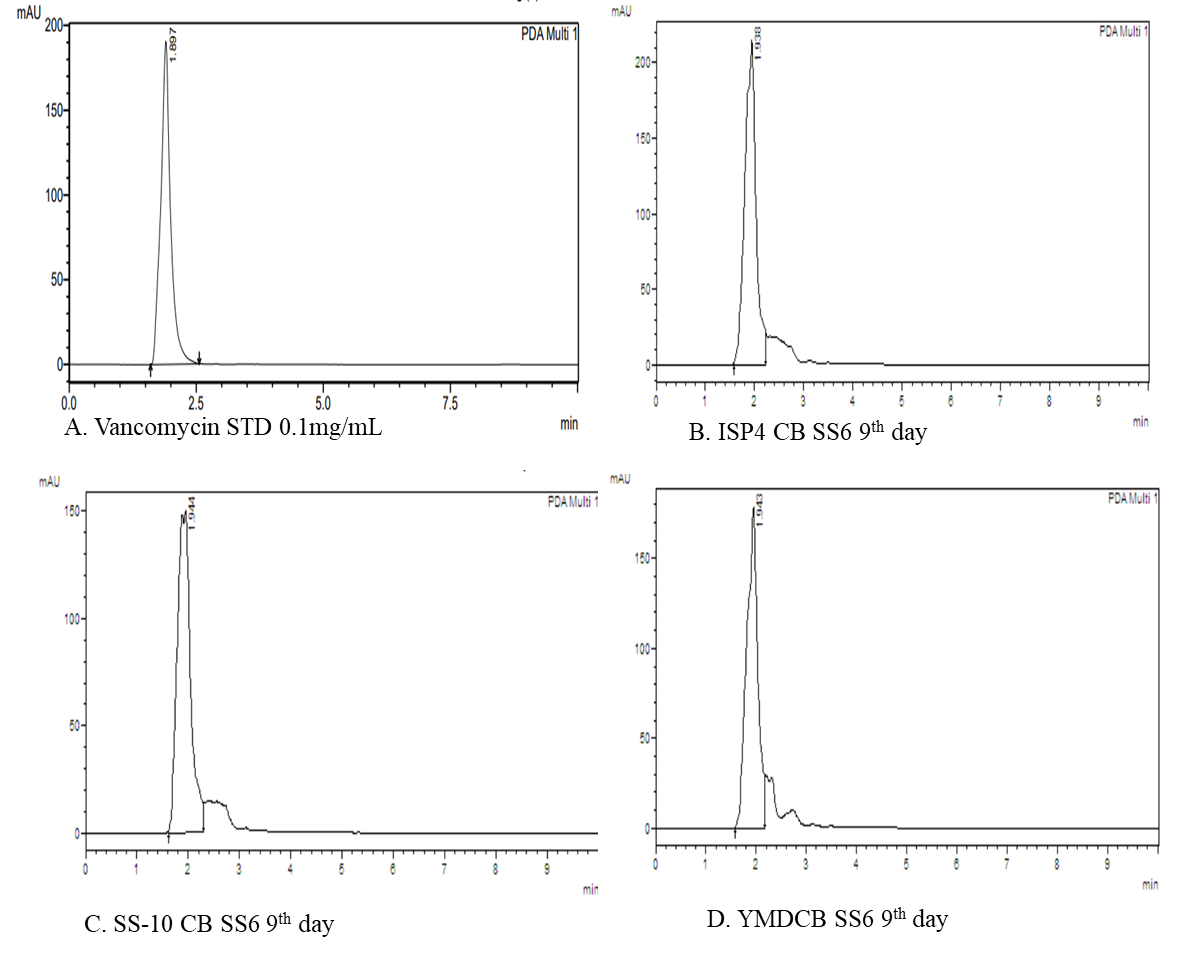


**Supplementary Figure 2.** HPLC analysis of A) Vancomycin STD 0.1mg/ml B) ISP4 CB SS69th day C) SS-10 CB SS6 9^th^ day and D) YMDCB SS6 9^th^ day
