# Supplementary material for: The Roles of Carbon‐Nitrogen Synergy and Phosphate Regulation in Producing Higher Yield of Vancomycin by Amycolatopsis orientalis
Source: Microbiologyopen. 2025 Oct 29;14(6):e70072. doi: 10.1002/mbo3.70072 (PMC12569529; doi:10.1002/mbo3.70072)
Supplement: Supplementary file 2 — Supplementary Table 1: Physical characteristics of A. orientalis in different types of media for spore growth. Supplementary Table 2: Physical characteristics of A. orientalis in different carbon sources. Supplementary Table 2: Physical characteristics of A. orientalis in different Nitrogen sources. Supplementary Table 4: Physical characteristics of A. orientalis in different seed media. [file MBO3-14-e70072-s001.docx]

**Supplementary Table 1.** Physical characteristics of *A. orientalis* in different types of media for spore growth.

| **Media Name** | **Colony Size** | **Colony Morphology** | **Spore Formation** | **Observed Plates** |
| --- | --- | --- | --- | --- |
| **ISP -1** | 2-3 mm | Wavy | +++ | 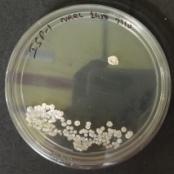 |
| **ISP-2** | 3-4 mm | Volcano- shaped | ++++ | 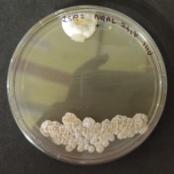 |
| **YMD** | 3-4 mm | Volcano-shaped, wavy | +++++ | 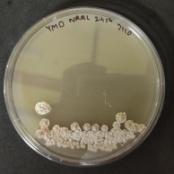 |
| **M65** | 3-4 mm | Volcano-shaped, wavy | +++++ | 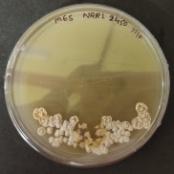 |
| **OM** | 3-4 mm | Volcano-shaped, wavy | +++++ | 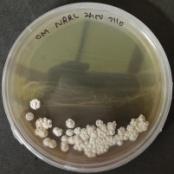 |

+++++: Excellent, ++++: Good, +++: Minimal

**Supplementary Table 2.** Physical characteristics of *A. orientalis* in different carbon sources.

| **S.no** | **Name of the media** | **Growth on plates** | **Observation on broth** |
| --- | --- | --- | --- |
| 1 | Carbon control | + | Less growth |
| 2 | Glucose | ++++ | Good growth |
| 3 | Fructose | ++++ | Good growth |
| 4 | Sucrose | +++ | Less growth |
| 5 | Sorbitol | +++ | Less growth |
| 6 | Starch | +++++ | Thick growth |
| 7 | Maltodextrin | +++++ | Good growth |
| 8 | Malt extract | +++++ | Good growth |
| 9 | glycerol | +++++ | Good growth |
| 10 | Skim milk | +++ | Less growth |
| 11 | Maltose | +++ | Less growth |

+++++: Excellent, ++++: Good, +++: Minimal, ++: Poor, +: Verypoor growth

**Supplementary Table 2.** Physical characteristics of *A. orientalis* in different Nitrogen sources

| Sl. no. | Nitrogen source | Growth on plates | Observation on plates | Observation on broth |
| --- | --- | --- | --- | --- |
| 1 | control | ++ | 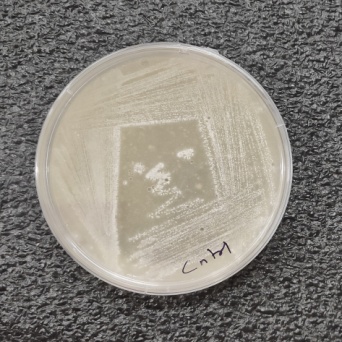 | Less growth |
| 2 | Soya peptone | +++ | 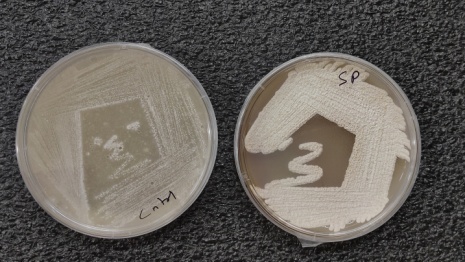 | Less growth |
| 3 | Soya bean meal | +++++ | 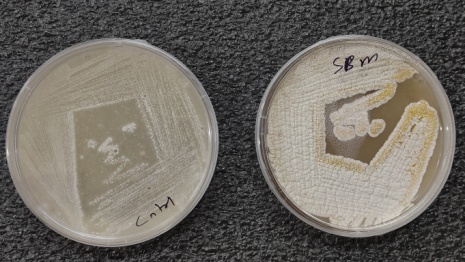 | Good growth |
| 4 | Soya flour | ++++ | 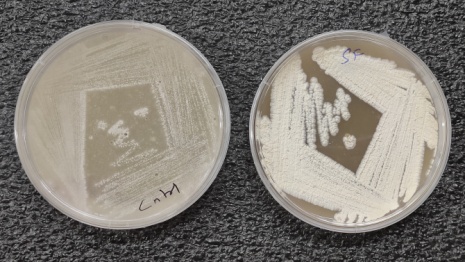 | Good growth |
| 5 | Yeast extract | +++++ | 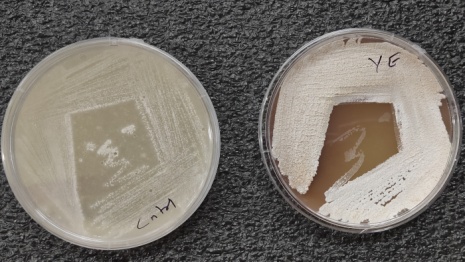 | Good growth |
| 6 | Tryptone | ++++ | 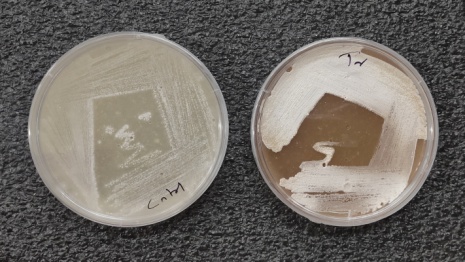 | Good growth |
| 7 | Cotton seed flour | +++ | 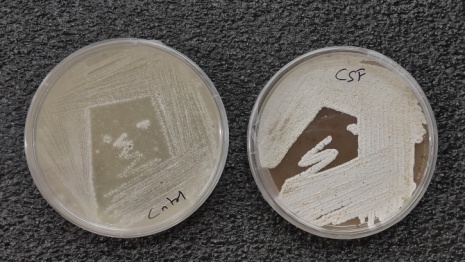 | Less growth |
| 8 | Ammonium sulphate | + | 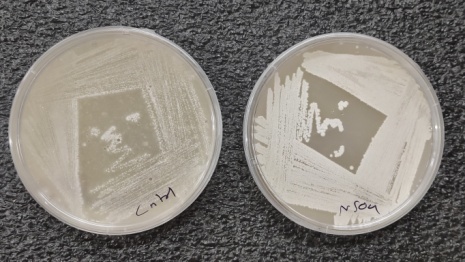 | Less growth |
| 9 | Potassium nitrate | ++ | - | Less growth |
| 10 | Sodium nitrate | + | - | Less growth |

**Supplementary Table 4. Physical characteristics of *A. orientalis* in different seed media.**

| **Media name** | **Characteristics** | **Colour** |
| --- | --- | --- |
| ISP-2 | Aggregated Pellets | Yellow |
| SS-3 | Mycelial/less pellet | Pale Yellow |
| SS-4 | Pellet mycelia | Yellow |
| SS-5 | Mycelial/Large pellet | Yellow |
| SS-6 | Large Pellet | Green Blue |
| SS-7 | Mycelial | Yellow |
| Seed-1 | Pellets | Yellow |
| Seed-2 | Small pellet | yellow |
| Seed-3 | Small pellets | Yellow |
| Seed-4 | Less pellet | Clear broth |
